# Enzyme stability in polymer hydrogel–enzyme hybrid nanocarrier containing phosphorylcholine group†

**DOI:** 10.1039/d4ra02436b

**Published:** 2024-06-11

**Authors:** Xuejin Huang, Jincai Li, Yasuyuki Araki, Takehiko Wada, Yan Xu, Madoka Takai

**Affiliations:** a Department of Bioengineering, School of Engineering, The University of Tokyo 7-3-1, Hongo, Bunkyo-ku 113-8656 Tokyo Japan takai@bis.t.u-tokyo.ac.jp; b Institute of Multidisciplinary Research for Advanced Materials, Tohoku University Sendai Japan; c Department of Chemical Engineering, Graduate School of Engineering, Osaka Metropolitan University Sakai Osaka Japan

## Abstract

Enzymes are biological catalysts with good biocompatibility and high efficiency and have been widely used in many fields, such as wastewater treatment, biosensors, and the medical industry. However, their inherently low stability under conditions of practical use limits further applications. Zwitterionic polymers possessing a pair of oppositely charged groups in their repeating units can increase protein stability because of their good biocompatibility and high water content. In this study, zwitterionic copolymer nanogels comprising poly(2-methacryloyloxyethyl phosphorylcholine (MPC)-*co*-methacrylic acid-*N*-hydroxy succinimide ester (MNHS)) (PMS) were synthesized *via* reversible addition–fragmentation chain-transfer polymerization (RAFT). β-Galactosidase (β-gal) was post-modified within zwitterionic polymer nanogels with a covalently-bound spacer and the activity was compared with that of directly immobilized β-gal and free β-gal. Compared with direct immobilization, covalent immobilization with a spacer could reduce the structural change of β-gal, as confirmed by the circular dichroism spectra. Although the activity of β-gal decreased after immobilization, the hybrids of the β-gal immobilized nanogels, termed hybrid nanogel–enzymes, demonstrated superior stability compared to the free enzymes. The hybrid nanogel–enzymes maintained their function against inactivation by organic solvents and proteinases owing to their high water content, anti-biofouling properties, and limited mass transfer. They can also withstand protein aggregation at high temperatures and maintain their activity. Compared to direct immobilization, immobilization with a spacer resulted in a dramatic increase in the enzyme activity and a slight decrease in the stability. These results indicate that polymer nanogels containing phosphorylcholine units are promising materials for enzyme immobilization, expanding the scope of enzyme applications.

## Abstract 2

Immobilization with a spacer could improve retained enzyme activity compared with direct immobilization. Zwitterionic polymer nanogels could enhance enzyme stability under various harsh conditions such as high temperature, and organic solvent.

## Graphical abstract

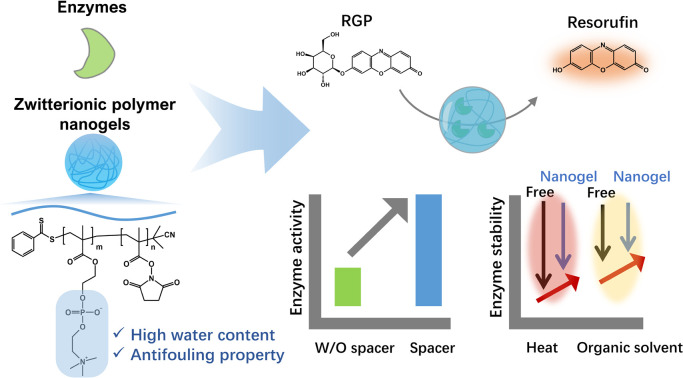

## 1. Introduction

Enzymes are biological catalysts with high activity and selectivity and are able to catalyze numerous reactions under mild conditions. This ability has made them valuable in many fields, such as wastewater treatment,^1^ biosensors,^2^ and the food industry.^3^ One major challenge in the industrial application of enzymes is their poor stability. Unlike physiological environments, practical application conditions can be extreme due to high temperatures and organic solvents, as well as protein denaturants, which can have a harmful and even irreversible impact on enzyme activity.^4^

Tremendous efforts have been made to improve the enzyme stability. This can be achieved by modifying the enzyme structure,^5–7^ adjusting the microenvironment surrounding the enzyme,^8,9^ and immobilizing them on or within support materials.^10–13^ Among these methods, enzyme immobilization is simple, cost-effective, provides good control over the microenvironment around the immobilized enzymes, and is commonly used to improve enzyme stability.^14^

However, immobilization may have undesirable effects on enzyme properties, such as limiting mass transfer and inducing conformational changes due to the interaction between the enzyme and the material, leading to severe activity loss.^15,16^ Therefore, selecting a suitable support material (carrier) and immobilization strategy is crucial for ensuring adequate performance of immobilized enzymes.^17,18^

Enzyme immobilization strategies can be categorized as physical adsorption,^19^ entrapment,^20^ and covalent binding.^21^ Physical adsorption and entrapment can minimize the unfavorable interactions between enzymes and materials, thereby preserving high enzymatic activity. However, these methods have the drawback that enzymes can easily leak from the material. Covalent immobilization can prevent enzyme leakage due to the formation of covalent bonds between the enzymes and materials. However, this may lead to significant structural changes and severe activity loss of the immobilized enzymes.

Introducing a spacer between the carrier and enzyme is one possible method of overcoming the drawbacks caused by covalent immobilization. The spacer could reduce unfavorable interactions with the carrier and reduce the steric hindrance between the immobilized enzymes and the substrate.^22,23^ The spacer arm can also facilitate the interaction between the functional groups on the support and enzymes by allowing access to protein groups far from the carrier surface.^24^ Many molecules, such as glutaraldehyde, 1,6-hexanediol diglycidyl ether, and ethylenediamine, have been used as spacers to facilitate enzyme immobilization, and it has been proven that immobilization with a spacer can improve the retained activity compared with direct immobilization.^25,26^

The carrier material is another factor that must be considered during the immobilization of enzymes. A good carrier should have good biocompatibility with enzymes without impairing the enzyme activity, and porous structures to facilitate the transport of substrates to the immobilized enzymes.^27^ Polymer hydrogels are crosslinked hydrophilic polymer networks with porous structures and high water content, which enables small molecules to penetrate the structure.^28,29^ Zwitterionic molecules, which contain both cationic and anionic units, could function as natural osmolytes in organisms, similar to sugar beets or saline soul herbs, thereby enhancing protein stability.^30–32^ Zwitterionic hydrogels, which possess the unique properties of both hydrogels and zwitterions, have been used to encapsulate enzymes to achieve enhanced activity and stability.^33,34^ Compared with bulk hydrogels, nanosized hydrogels, with diameters ranging from tens to hundreds of nanometers, are prospectively superior materials for enzyme immobilization because their extensive surface area. Such materials can enhance the reaction rate, offering a significant advantage over bulk materials.^35,36^

**Fig. 1:**
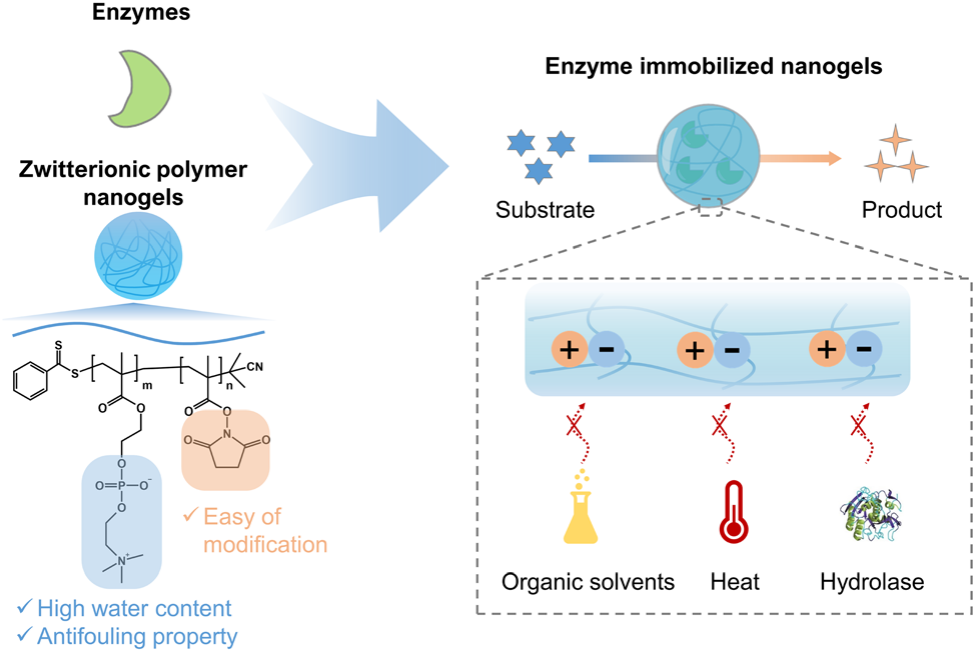
Illustration of zwitterionic nanogels for enzyme immobilization with or without a spacer to enhance the stability of the enzyme under various extreme conditions.

Recently, we developed enzyme-loaded zwitterionic polymer hydrogels composed of poly(2-methacryloyloxyethyl phosphorylcholine (MPC)-*co*-methacrylic acid-*N*-hydroxy succinimide ester (MNHS)) (PMS), where the immobilized enzymes exhibited long-term stability.^37^ Herein, we report several functions such as the heat stability, anti-organic solvent, and anti-hydrolase properties of enzymes within the nano-sized hydrogel of PMS, which is synthesized by reversible addition–fragmentation chain-transfer (RAFT) polymerization. The effect of immobilizing enzymes in polymer nanogels on the enzyme functions is evaluated by introducing spacers between the enzyme and polymer chain. An overview of this study is presented in Fig. 1. β-Gal, which can hydrolyze lactose to galactose and glucose, is selected as a model enzyme because of its widespread use in the food industry.^38^ The widespread use of β-gal calls for strategies to extend its shelf-life, enhance reusability, and improve structural stability.

## 2. Materials and methods

### 2.1 Materials

The following commercial chemicals were used as received unless otherwise noted. 2-Methacryloyloxyethyl phosphorylcholine (MPC) was purchased from NOF Corporation (Kawasaki, Japan). Methacrylic acid-*N*-hydroxysuccinimide ester (MNHS) and 2-cyanopropan-2-yl benzodithioate (CPB) were purchased from Sigma-Aldrich (St. Louis, MO, USA). Chloroform, ethanol, methanol, acetonitrile, dimethyl sulfoxide, α,α′-azobisisobutyronitrile (AIBN), 0.1 M phosphate buffer solution (PBS, pH 7.4), ethylenediamine (EDA), 1,4-butanediol diglycidyl ether (BDDE), guanidium chloride (GdmCl), proteinase K recombinant, and Protein Assay Micro-BCA Kit were purchased from FUJIFILM Wako Pure Chemical Corporation (Osaka, Japan). β-Galactosidase (>50 U mg^−1^) was purchased from MP Biomedicals, Inc. (Irvine, CA). Resorufin β-d-galactopyranoside (RGP) was purchased from Santa Cruz Biotechnology (California, US).

### 2.2 Synthesis of zwitterionic copolymer poly(MPC-*co*-MNHS) by RAFT polymerization

The zwitterionic copolymer, poly(MPC-*co*-MNHS) (PMS), with an equal monomer ratio was synthesized as described in our previous report.^37^ The procedure for RAFT polymerization is shown in Fig. S1.† In brief, a stock solution containing MPC (885.8 mg, 3 mmol), MNHS (550 mg, 3 mmol), AIBN (5 mg, 0.03 mmol), and the chain transfer agent CPB (13 mg, 0.06 mmol) in 6 ml ethanol/chloroform (v/v = 4/6) was thoroughly deoxygenated by argon bubbling for 30 min. The polymerization proceeded at 59 °C for 24 h. After polymerization, the PMS copolymer was reprecipitated with excess chloroform and vacuum-dried to remove residual chloroform.

### 2.3 Preparation of hybrid nanogel–enzymes

**Fig. 2:**
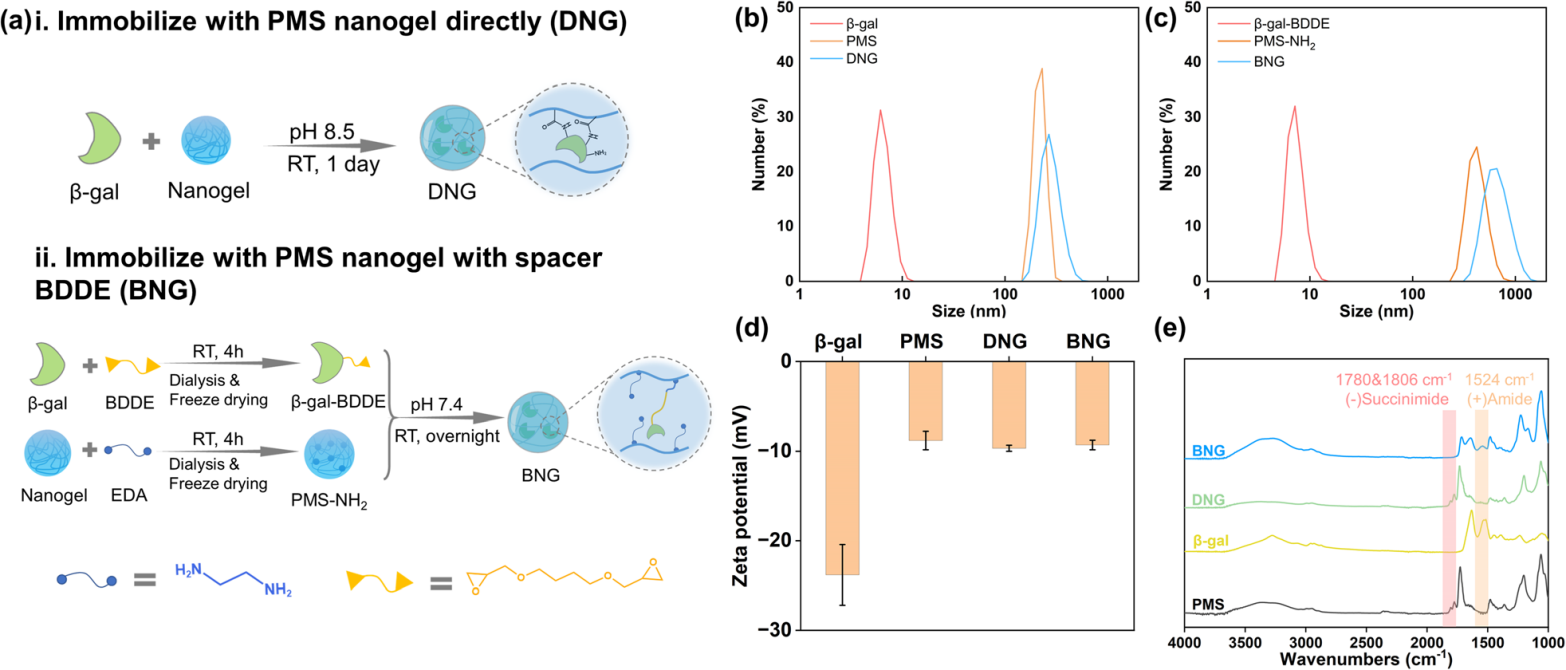
(a) Preparation of DNG (i) and BNG (ii); dynamic light scattering analysis of size distribution of DNG (b) and BNG (c); (d) zeta potential of β-gal, PMS, DNG, and BNG; (e) FTIR spectra of PMS, β-gal, DNG, and BNG.

To investigate the effect of the spacer on the properties of the enzyme after immobilization, β-gal was immobilized within PMS nanogels with the spacer BDDE and compared with directly immobilized enzymes. Direct immobilization utilizes the interaction between the primary amine groups on β-gal and succinimide groups on the nanogels, as shown in Fig. 2a(i). In brief, 1 mg ml^−1^ β-gal dissolved in 50 mM borate buffer (pH 8.5) was added to PMS powder with constant agitation to achieve a concentration 10 mg ml^−1^. Immobilization was performed at room temperature for one day. The products were prewashed through centrifugation and redispersion cycles (14 000 rpm, 15 min) to separate the unreacted enzymes; these products are denoted as DNG. The preparation process for immobilization with the spacer is shown in Fig. 2a(ii). Generally, PMS and β-gal must first be modified. PMS (100 mg) was firstly reacted with 10 μl ethylenediamine (EDA) in 10 ml PBS (pH 7.4) to endow it with amine groups (PMS-NH_2_), and 10 mg ml^−1^ β-gal was interacted with 5% 1,4-butanediol diglycidyl ether (BDDE) in PBS buffer for 4 h to modify β-gal with epoxy groups (β-gal-BDDE). The modified nanogel and enzyme were purified by dialysis in DI water (MWCO 14 kDa membrane, 1 day) and freeze-dried to obtain the products. The hybrid nanogel–enzymes were prepared by dissolving PMS-NH_2_ (10 mg ml^−1^) and β-gal-BDDE (1 mg ml^−1^) in PBS buffer and reacting at room temperature overnight. The hybrid nanogel enzymes were separated by centrifugation (14 000 rpm, 15 min) and named BNG.

### 2.4 NMR characterization

It's difficult for PMS to dissolve completely in D_2_O because it gelates together. Therefore, PMS copolymers were hydrolyzed completely in 0.1 M NaOH and dialyzed in deionized water to obtain poly(MPC-*co*-MMA). The composition of PMS can be indirectly obtained by detecting the molar ratio of poly(MPC-*co*-MMA) by high-resolution ^1^H NMR spectroscopy (JEOL ECS-400) using D_2_O as the solvent.

### 2.5 Dynamic light scattering and zeta potential

Dynamic light scattering (DLS) measurements were performed using a Zetasizer Pro ZSU3200 instrument (Malvern Instruments, Malvern, U.K.). PMS and the hybrid nanogel enzymes were centrifuged under 14 000 rpm before measuring their size; the hydrodynamic radius and polydispersity index (PDI) were obtained using ZS XPLORER software. Zeta potential measurements were also performed using electrophoretic light scattering (ELS).

### 2.6 Fourier-transform infrared spectroscopy

Formation of the hybrid nanogel enzymes was analyzed by Fourier-transform infrared (FT-IR) spectroscopy using an IRSpirit-T spectrometer (SHIMADZU Corp, Japan).

### 2.7 Field-emission scanning electron microscopy

The morphology of the prepared hybrid nanogel–enzymes was observed using field-emission scanning electron microscopy (FE-SEN S4200, 15 kV, Hitachi, Japan).

### 2.8 Circular dichroism spectroscopy

Secondary structural changes in the enzyme after immobilization were detected in deionized water by circular dichroism spectroscopy at room temperature using a JASCO J-820 spectropolarimeter. The change in the secondary structure of the enzyme samples at high temperatures was detected in 0.1 M PBS solution. Data were collected in steps of 1 nm, covering the spectral range of 350–200 nm; the path-length was 10 mm. The spectra were averaged over four consecutive scans, and the solvent signal without enzymes was subtracted.

### 2.9 Fluorescence spectroscopy

β-Gal solution (3 ml; 10 μg ml^−1^) in 0.1 M PBS buffer was placed in a quartz cuvette. The emission spectra were recorded from 300 to 450 nm using a JASCO FP-6600 spectrofluorometer with an excitation wavelength of 280 nm.

### 2.10 Enzymatic activity

The enzymatic activity was evaluated by utilizing the principle that resorufin β-d-galactopyranoside (RGP) can be hydrolyzed by β-gal to yield fluorescent resorufin. The Michaelis–Menten constants, *K*_m_ and *V*_max_, for both free and immobilized β-gal, were measured by tracking the fluorescence change over time with varying concentrations of the RGP solution. The assay was conducted by adding 100 μl of each sample (3.7 nM) to a well of a 96-well plate in triplicate. This was immediately mixed with 100 μl of RGP solution with varying concentrations and the change in the fluorescence intensity was monitored using a plate reader. The excitation and emission wavelengths were 560 and 595 nm, respectively. The fluorescence intensity was converted to the concentration of resorufin produced by comparison with a calibration curve correlating the resorufin concentration and fluorescence intensity.

### 2.11 Proteinase digestion test

Free β-gal, DNG, and BNG (3.7 nM) were incubated with 250 μg ml^−1^ proteinase K solution at 37 °C and the residual activity was measured to investigate the resistance of the zwitterionic nanogel against proteinase degradation. The time at which proteinase was added was recorded as 0 min.

### 2.12 Stability test

The stability against organic solvents was measured by mixing 90 μl of the sample (3.7 nM) with 10 μl of the organic solvent (methanol, acetonitrile, and dimethyl sulfoxide) and incubating for 30 min at room temperature. The mixture was then placed in a well of 96-well plate to measure the residual activity. To evaluate the thermal stability, free β-gal, DNG, and BNG were annealed at 65 °C in an oven. Samples were withdrawn at 5, 15, 30, 45, and 60 min. The residual activities were measured at room temperature by adding 20 μM RGP solution and compared with their original activities (0 min). All tests were performed in triplicate.

### 2.13 Statistic analysis

Data were presented in the form of mean ± standard deviation. All tests were conducted in triplicate.

## 3. Results and discussion

### 3.1 Characterization of hybrid nanogel–enzymes

PMS was synthesized by RAFT polymerization and characterized by ^1^H NMR spectroscopy (Fig. S2†) and GPC. The results listed in Table S1† show that the molar ratio of MPC to MNHS in PMS (*M*_n_ = 12 200) was 57 : 43, which is in good agreement with the feed ratio (50 : 50). PMS nanogels can be obtained by centrifugation and its size depends on its molecular weight (Fig. S3†). To immobilize β-gal inside nanogels, we adopt a similar method from previous research that introduces enzyme solutions to the PMS powder to allow enzymes to be absorbed into nanogels *via* capillary action during the swelling process.^39^ The covalent bonds between absorbed enzymes and nanogels could prevent enzyme leakage. To investigate the effect of the spacer on the enzyme functions, β-gal was immobilized within PMS nanogels with a spacer BDDE. Though one-pot method could be a simple and efficient method to immobilize enzymes, the complex environment may denature enzymes, leading to a significant activity loss (Fig. S4†). The formation of hybrid nanogel–enzymes was confirmed by DLS, FE-SEM, zeta-potential, and FT-IR analyses. The changes in the size of the raw materials and products before and after immobilization were analyzed. The size of DNG increased to ∼270 nm after interacting with β-gal and the size of BNG changed to ∼680 nm after immobilization (Fig. 2b and c), which is in accordance with the SEM observation that BNG has a larger size than DNG (Fig. S5†). The hydrophobic moiety, MNHS, plays a crucial role in the compaction of the physically crosslinked PMS nanogel because hydrophobic interactions contribute significantly to the stability of the nanogel structure. However, when MNHS is substituted with a hydrophilic compound, such as ethylenediamine, the nanogel swells owing to disruption of the internal hydrophobic interactions, leading to an increase in particle size. Fig. 2d displays the change in the zeta potential of free and immobilized β-gal. The zeta potential of β-gal increased from −23.8 mV to −9.7 mV for DNG and −9.3 mV for BNG because the surface charge of the protein was shielded by PMS. Fig. 2e shows the FT-IR spectra of PMS, β-gal, DNG, and BNG. The succinimide ester groups in the nanogels can interact with the primary amine groups of β-gal under alkaline conditions and generate stable amide groups. The spectrum of PMS exhibits characteristic signals at 1780 and 1806 cm^−1^, which originate from the succinimide groups.^40^ However, these signals disappeared in the spectra of BNG and were of slightly lower intensity in the spectra of DNG, indicating that the succinimide ester groups were replaced by other groups. A new peak was observed at 1524 cm^−1^ in the spectra of BNG and DNG, corresponding to the newly formed amide groups. These results demonstrate the successful formation of the hybrid nanogel–enzymes.

### 3.2 Kinetics of hybrid nanogel–enzymes

**Fig. 3:**
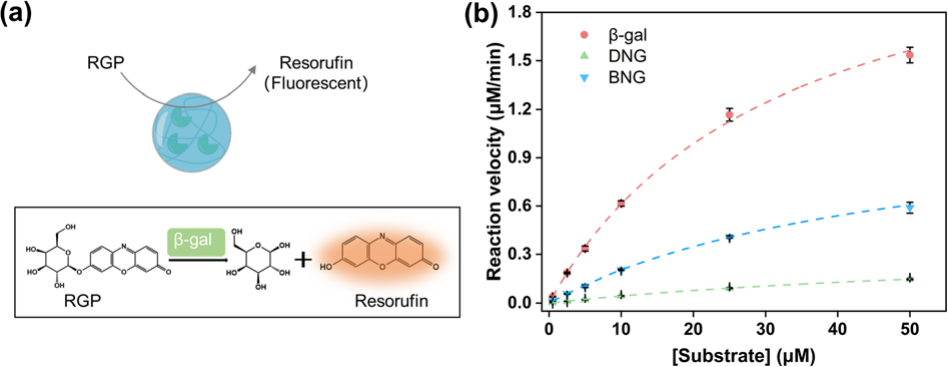
(a) Illustration of enzymatic reactions of hybrid nanogel–enzymes during the hydrolysis of RGP to yield resorufin; (b) Michaelis–Menten kinetics for β-gal, DNG, and BNG at pH 7.4.

To investigate the effect of immobilizing the PMS nanogels, the reaction kinetics of the hybrid nanogel–enzymes were studied using RGP as the substrate, which can be hydrolyzed by β-gal to generate resorufin (Fig. 3a). The fluorescence intensity was linearly related to the resorufin concentration (Fig. S6†). Therefore, the detected fluorescence intensity could be converted to the concentration of the generated resorufin, and the catalytic velocity with varying substrate concentrations could be calculated. Kinetic parameters such as *K*_m_, *V*_max_, and *k*_cat_ were obtained by fitting the reaction velocity to the Michaelis–Menten model (Fig. 3b). These parameters are summarized in Table 1, demonstrating that the enzymatic activity of immobilized β-gal decreased compared to that of the free enzyme (*k*_cat_ = 11.53 s^−1^). Notably, for DNG (*k*_cat_ = 1.67 s^−1^), a 7-fold decrease was observed. The decrease in the catalytic performance might be associated with the restriction of substrate and product diffusion to and from the PMS nanogels given that *K*_m_ was larger for BNG and DNG than for free β-gal. *K*_m_ is an indicator of the affinity between the substrate and enzymes, where a larger *K*_m_ indicates a lower affinity between the enzyme and the substrate. Generally, immobilization of enzymes on materials with a spacer could improve the retained activity compared to immobilization directly on it. A longer spacer typically could enhance enzymatic activity by increasing the substrate affinity^23^ and the flexibility of immobilized enzymes.^41^ Compared to DNG, BNG exhibited improved catalytic performance because the spacer reduced the steric hindrance between the immobilized enzyme and the substrate to facilitate the catalytic interaction.^42^ This is evidenced by the smaller *K*_m_ of BNG, indicating a higher affinity between BNG and the substrate.

**Table 1:** Kinetic parameters for free and immobilized β-gal from fitting to the Michaelis–Menten model

Samples	β-Gal	DNG	BNG
*K*_m_ (μM)	31.77 ± 1.37	74.36 ± 7.88	49.46 ± 3.53
*V*_max_ (μM min^−1^)	2.56 ± 0.09	0.37 ± 0.03	1.21 ± 0.07
*k*_cat_ (s^−1^)	11.53	1.67	5.45

**Fig. 4:**
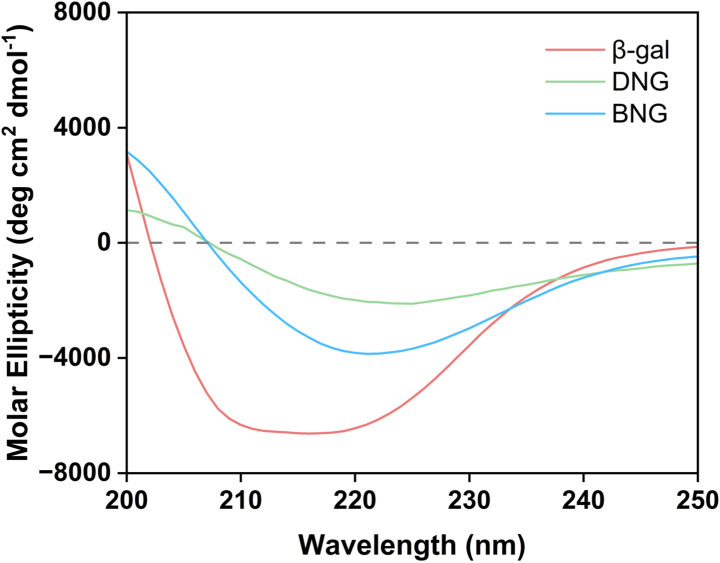
Circular dichroism spectra of β-gal, DNG, and BNG in DI water.

Besides limiting mass transfer, changes in the enzyme structure upon immobilization may also contribute to activity loss. To confirm our hypothesis that the decrease in the activity of immobilized β-gal was associated with the conformational change, the structure was analyzed using circular dichroism spectroscopy. Fig. 4 displays the secondary structures of free and immobilized β-gal in the far-UV spectral region (200–250 nm). The spectrum of β-gal showed the typical negative signals of the α-helix structure at 208 and 222 nm. The signal amplitude decreased at this wavelength for the hybrid nanogel–enzymes compared with that of free β-gal, indicating that immobilization caused a structural change. The loss of negative ellipticity for DNG was more significant than that for BNG, indicating that DNG underwent greater conformational changes. This result demonstrates that the spacer could also help reduce secondary structural loss and increase the retained activity after immobilization.

### 3.3 Proteinase degradation test

**Fig. 5:**
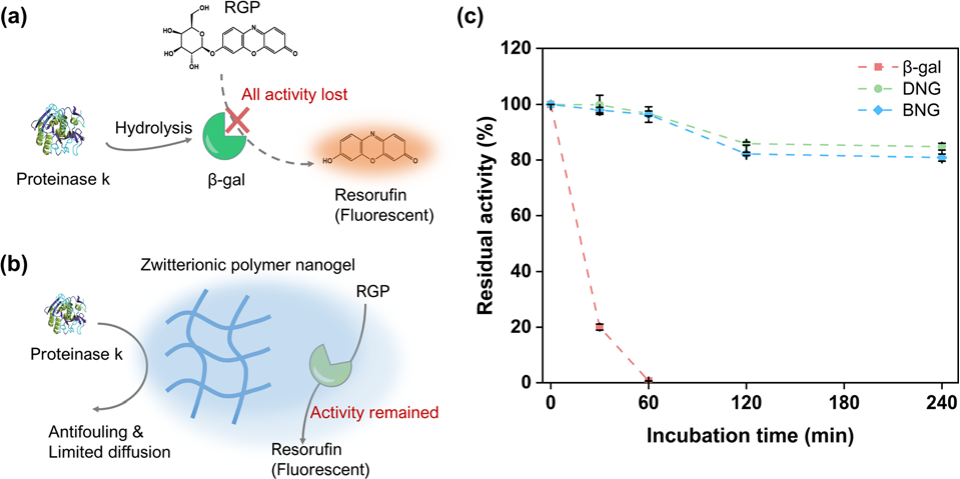
Illustration of the hydrolysis of β-gal by proteinase K (a) and the protection of immobilized β-gal inside zwitterionic nanogels from proteinase degradation (b); (c) residual activity of β-gal, DNG, and BNG after incubation with 250 μg ml^−1^ proteinase at 37 °C for different times.

To verify whether the enzymes were immobilized inside or outside the zwitterionic nanogels, the hybrid nanogel–enzymes were incubated with proteinase K, a protein hydrolase that can hydrolyze free enzymes. Preservation of the enzymatic activity is contingent on encapsulation of the enzymes within the zwitterionic nanogels, attributed to the antifouling properties of MPC and restricted diffusion facilitated by the polymeric network. These factors collectively inhibit interactions between the proteinases and encapsulated enzymes. As shown in Fig. 5c, free β-gal quickly lost its activity within the first 30 min and completely lost its activity after 1 h incubation, which is reasonable because free β-gal would be exposed to the high concentration of proteinase (Fig. 5a). In contrast, immobilization within the zwitterionic nanogels could protect β-gal from degradation by proteinase. The activities of DNG and BNG decreased slowly, where approximately 80% of the activity was maintained after 240 min of incubation, attributed to the antifouling property and polymeric network of the PMS nanogels. The antifouling properties of the MPC moieties can prevent non-specific protein adsorption, and the polymeric network of the PMS nanogels can limit the diffusion of proteinase K to the immobilized β-gal, therefore preserving the activity (Fig. 5b).

To confirm our hypothesis that the polymeric network of nanogels could limit the internal diffusion of proteinase and protect the immobilized enzymes from proteinase degradation, guanidium chloride (GdmCl) was used as a small molecular denaturant to deactivate encapsulated β-gal. GdmCl can penetrate through the polymer network into the nanogels and denature encapsulated β-gal (Fig. S7a†). DNG and BNG lost more than 80% of their activity after 30 min incubation (Fig. S7b†). However, they still maintained higher activity than free β-gal. This is because GdmCl cannot diffuse as freely in the nanogels as in the bulk solution. The polymeric network restricted the full access of GdmCl to the immobilized enzyme. These results indicate that although immobilization within nanogels may limit transport of the substrates, it can protect the immobilized enzymes from degradation by larger molecular denaturants such as proteinase.

### 3.4 Stability test

One advantage of enzyme immobilization is that it improves the stability of enzymes in extreme environments to meet the needs of industrial applications. Therefore, the stability of hybrid nanogel–enzymes was examined under different conditions.

**Fig. 6:**
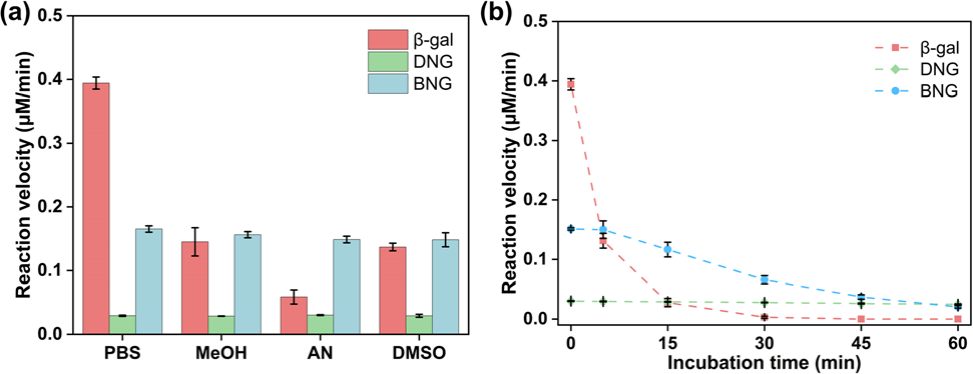
Stability of free and immobilized β-gal under different conditions. (a) Reaction velocities of β-gal, DNG, and BNG incubated with 10% (v/v) organic solvent (MeOH: methanol, AN: acetonitrile, and DMSO: dimethyl sulfoxide) and PBS buffer (pH 7.4, control sample); (b) thermal stability of β-gal, DNG, and BNG in 0.1 M PBS buffer incubated at 65 °C for 1 h.

Many enzymes need to function in organic media because the non-aqueous environment has several advantages, including increased solubility of non-polar substrates, shifting of the thermodynamic equilibrium to favor synthesis over hydrolysis, and inhibition of many water-dependent side reactions.^43^ However, these advantages are often limited by the low stability and activity of enzymes in organic solvents. Therefore, it is necessary to enhance the activity and stability of enzymes in organic solvents. As displayed in Fig. 6a, the activity of free β-gal decreased significantly after adding organic solvents; more than 50% of the activity was lost after incubation with 10% MeOH, AN, and DMSO. The possible mechanism for the activity loss may be related to the hydration layer on the enzyme surfaces. Water molecules are hydrogen-bonded to the protein surface, forming a hydration layer that is critical for enzyme function. However, the displacement of bound water by organic solvents can lead to drastic changes in the protein structure, resulting in loss of activity.^44^ Zwitterions have a high water content due to the ionic solvent effect, which could prevent stripping of the water layer from the protein surface and help preserve the activity of the enzyme in organic solvents.^45,46^ As a result, both DNG and BNG can help maintain enzyme activity in various organic media, such as MeOH, AN, and DMSO.

**Fig. 7:**
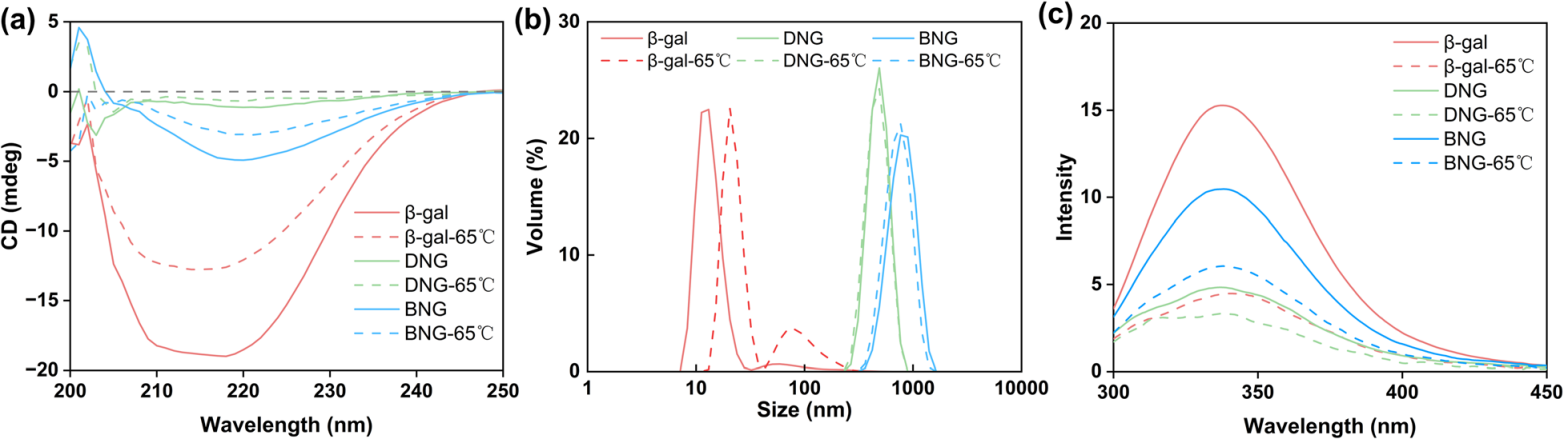
(a) CD spectra of β-gal, DNG, and BNG in 0.1 M PBS solution before and after annealing at 65 °C for 1 h. Phosphate buffer has a signal between 200 and 210 nm, interfering with the signals of enzymes at this wavelength. (b) Dynamic light scattering analysis and (c) intrinsic tryptophan fluorescence spectra of β-gal, DNG, and BNG in 0.1 M PBS buffer before and after annealing at 65 °C for 1 h.

Many mesophilic enzymes have poor activity and stability at high temperatures, limiting their industrial applications. Elevated temperatures are usually needed to increase productivity and reduce bacterial contamination.^44^ Simple and cost-efficient methods of improving the thermal stability of enzymes at high temperatures are thus necessary. Here, the thermal stability of hybrid nanogel–enzymes was tested at 65 °C; the results are presented in Fig. 6b. All the samples were annealed at 65 °C and the enzyme activity was measured at room temperature. The results showed a substantial difference in the residual activity of free and immobilized β-gal. The activity of free β-gal decreased dramatically after incubation at 65 °C for 15 min and was completely lost after 30 min. This is because at high temperatures, the proteins change from the folded state to the unfolded state and then aggregate due to the increased rate of protein diffusion.^47^ As shown in Fig. 7a, a dramatic decrease in the signal intensity in the CD spectra of β-gal was observed after incubation at 65 °C for 1 h, indicating a significant change in its secondary structure. The size of free β-gal increased after annealing at 65 °C for 1 h (Fig. 7b), suggesting that free β-gal formed aggregates during the heating process, leading to irreversible activity loss. Enzyme aggregation is associated with the structural change, as indicated by the significant decrease in the fluorescence intensity of tryptophan in free β-gal (Fig. 7c). Tryptophan often resides within the hydrophobic core of proteins. When the proteins change from the folded state to the unfolded state, tryptophan is exposed to the solvent, leading to a decrease in the intrinsic fluorescence intensity.^48^ Consequently, the decrease in the intrinsic fluorescence intensity can be interpreted as a change in the tertiary and quaternary structure of the enzyme. Compared with free β-gal, the thermal stability of the enzymes immobilized within the PMS nanogels was enhanced. Although the PMS nanogels may not entirely prevent the secondary and tertiary structural changes induced by heat (Fig. 7a and c), they can inhibit aggregation of the unfolded enzymes because the enzymes are immobilized within the nanogels. As shown in Fig. 7b, the size of DNG and BNG did not increase after incubation at 65 °C for 1 h. In the case of the immobilized enzymes, DNG and BNG displayed different trends. Compared with DNG, the enzyme activity of BNG decreased significantly after incubation for 1 h due to the severe structural change at high temperatures (Fig. 7a and c).

## 4. Conclusions

PMS with an equal feed ratio of MPC to MNHS was synthesized *via* RAFT polymerization and characterized by ^1^H NMR and GPC. β-Gal was immobilized within PMS nanogels *via* covalent immobilization with or without the BDDE spacer (BNG and DNG, respectively). The formation of the hybrids was validated by DLS, FE-SEM, and FTIR. The observed changes in the particle size, zeta potential, and functional groups confirmed the successful formation of the hybrid nanogel–enzymes. Kinetics studies revealed significant alterations in the enzyme activity upon immobilization. The *k*_cat_ values of DNG and BNG decreased by 7-fold and 2-fold, respectively. This reduction in the catalytic efficiency is attributed to restricted substrate transport within the immobilized system and loss of the secondary structure upon immobilization. Interestingly, the enzymatic activity of BNG was superior to that of DNG, which could be ascribed to the presence of the spacer. The spacer increases the distance between the immobilized enzyme and the polymer chain, which can inhibit structural changes and reduce steric hindrance to facilitate interactions between the enzymes and substrates. Although encapsulation in the PMS nanogels impaired the enzymatic activity, both DNG and BNG showed enhanced stability under extreme conditions. Immobilization within the PMS nanogels protected β-gal from degradation by proteinase K because the antifouling property of MPC inhibits the non-specific protein adsorption and the polymeric networks of the nanogel limit the diffusion of large molecular denaturants to the internal region. The PMS nanogel can also help maintain the function of immobilized β-gal in organic solvents by resisting stripping of the water molecules around the enzyme surface by organic solvents. In addition, the PMS nanogel improved the thermal stability of β-gal by reducing the conformational change and inhibiting protein aggregation at high temperatures. These results demonstrate that zwitterionic polymer nanogels containing phosphorylcholine units are prospectively suitable nanocarriers for enzyme immobilization and that the prepared hybrid nanogel–enzymes have tremendous potential for industrial applications that require harsh conditions.

## Data availability

The data that support the findings are available on request from the corresponding author.

## Conflicts of interest

There are no conflicts to declare.

## Supplementary Material

RA-014-D4RA02436B-s001
