# Supplementary material for: Enzyme stability in polymer hydrogel–enzyme hybrid nanocarrier containing phosphorylcholine group
Source: RSC Adv. 2024 Jun 11;14(26):18807–14. doi: 10.1039/d4ra02436b (PMC11166189; doi:10.1039/d4ra02436b)
Supplement: RA-014-D4RA02436B-s001 [file RA-014-D4RA02436B-s001.pdf]

Electronic Supplementary Material (ESI) for RSC Advances.
This journal is © The Royal Society of Chemistry 2024

Supporting Information

Enzyme stability in polymer hydrogel-enzyme hybrid nanocarrier containing phosphorylcholine group

Xuejin Huang,[a] Jincai Li,[a] Yasuyuki Araki,[b] Takehiko Wada,[b] Yan Xu,[c] Madoka Takai[a]

[a] Department of Bioengineering, School of Engineering, The University of Tokyo, 7-3-1, Hongo, Bunkyo-ku, 113-8656, Tokyo, Japan.
[b] Institute of Multidisciplinary Research for Advanced Materials, Tohoku University, Sendai, Japan
[c] Department of Chemical Engineering, Graduate School of Engineering, Osaka Metropolitan University, Sakai, Osaka, Japan.

List of Figures and Tables

Fig S1. Schematic illustration of the synthesis of PMS via RAFT polymerization

Fig S2. ^{1}H NMR of hydrolyzed PMS in D_2O

Table S1. Characterization of the synthesized PMS

Fig S3. Size of PMS with different polymerization degrees after centrifugation.

Fig S4. (a) Size of BNG synthesized by one-pot method; (b) comparison of the BNG activity synthesized by different methods.

Fig S5. FE-SEM images of hybrid nanogels DNG and BNG

Fig S6. The calibration curve shows the relationship between the fluorescence intensity and the concentration of resorufin

Fig S7. (a) Illustration of the denaturation of β-gal immobilized inside zwitterionic nanogels caused by guanidinium chloride; (b)Residual activity of β-gal, BNG and DNG incubated with 6M guanidinium chloride for 30 min.

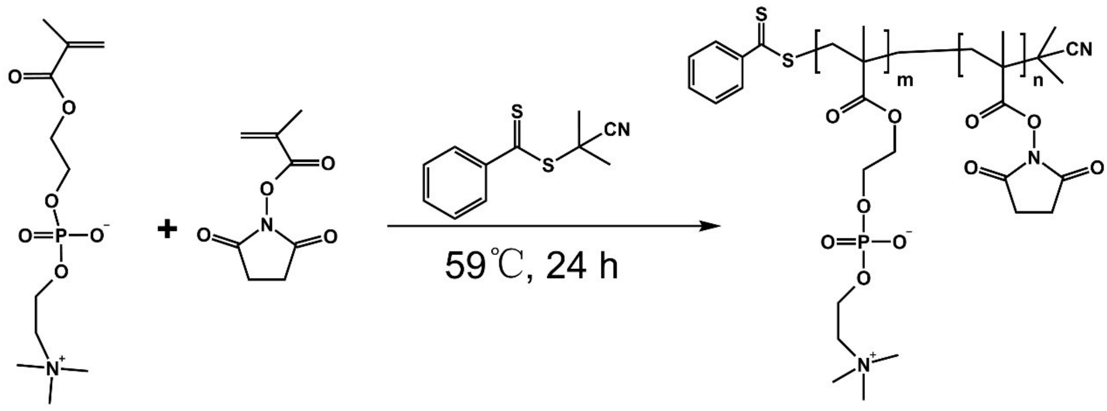

MPC MNHS PMS

Fig S1. Schematic illustration of the synthesis of PMS via RAFT polymerization

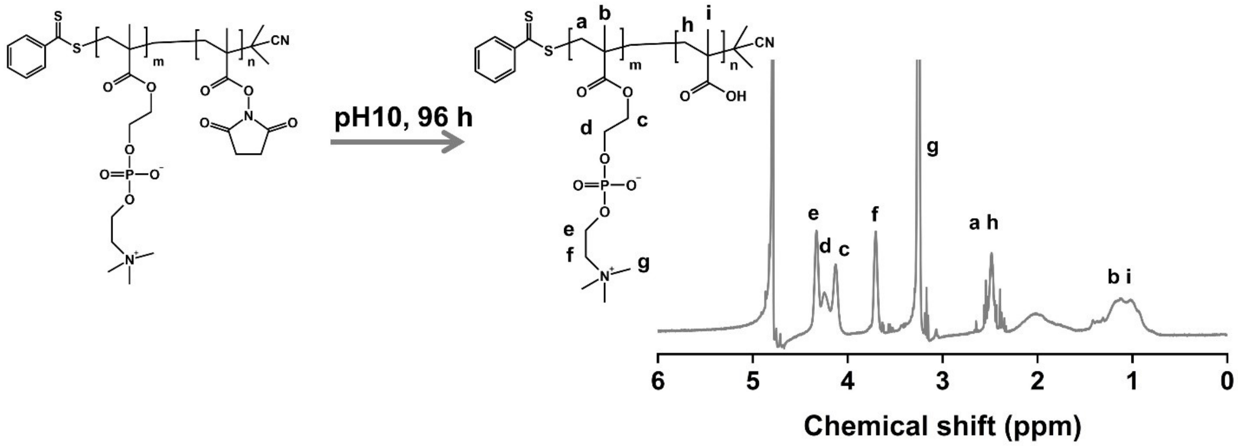

Fig S2. ^{1}H NMR of hydrolyzed PMS in D_2O. δ H (400 MHz, D2O) 4.33 (2H), 4.25 (2H), 4.13(2H), 3.70 (2H), 3.26 (9H), 2.49 (2H), 2.40 (2H), 1.13 (3H), 1.02 (3H).

Table S1. Characterization of the synthesized PMS

	Molar ratio in feed MPC/MNHS	Molar ratio in precursor [a] MPC/MNHS	Mn [b]	Mw/Mn [b]
PMS	50/50	57/43	12200	1.37

a. Determined by ^{1}H NMR in D_2O
b. Detected by GPC

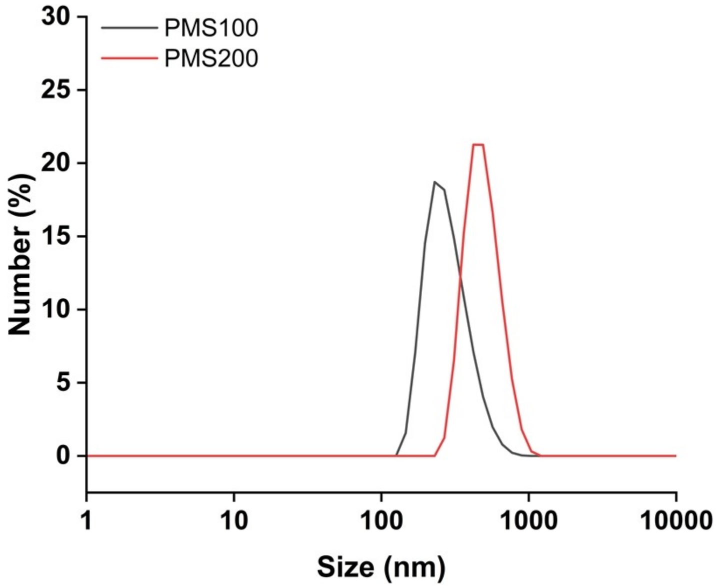

Fig S3. Size of PMS with different polymerization degrees after centrifugation (PMS100 and PMS200 mean their polymerization degree are 100 and 200, respectively).

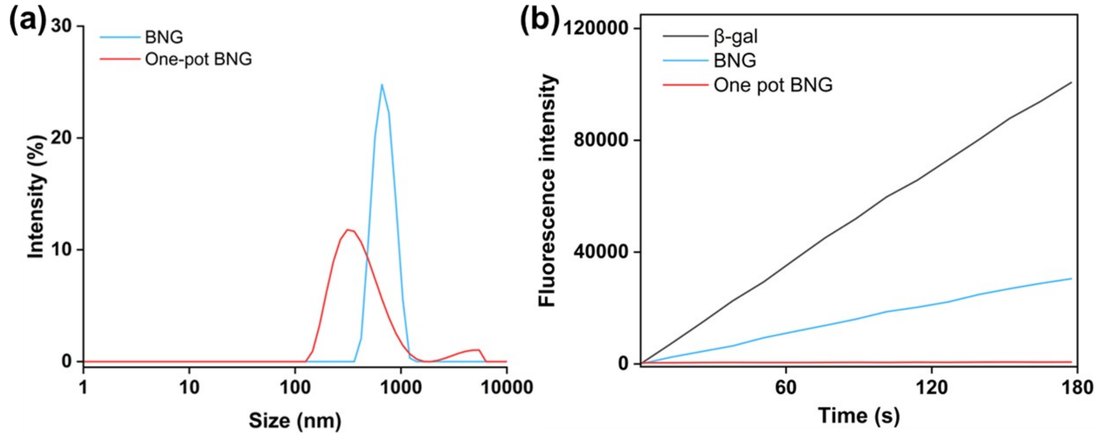

Fig S4. (a) Size of BNG synthesized by one-pot method; (b) comparison of the BNG activity synthesized by different methods.

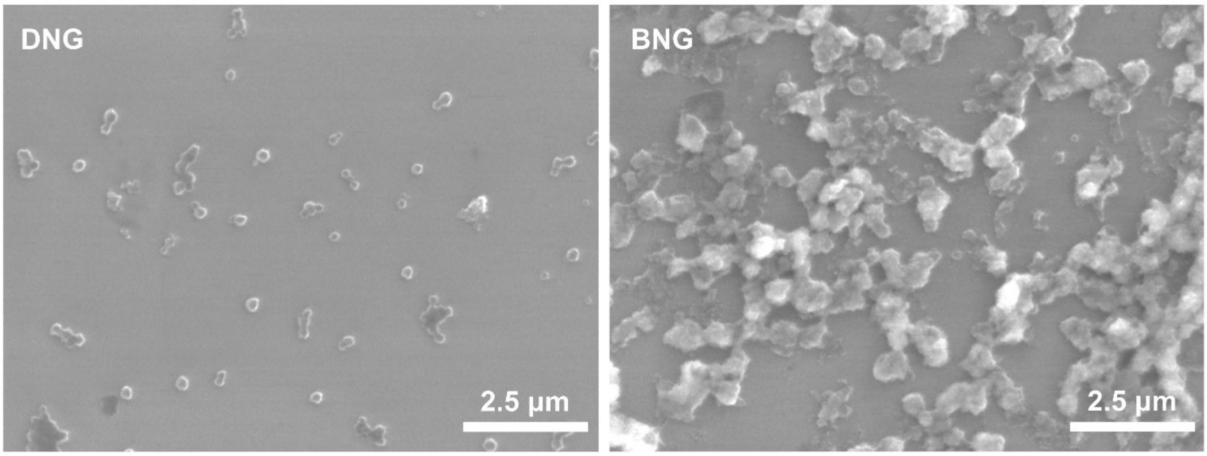

Fig S5. FE-SEM images of hybrid nanogels DNG and BNG.

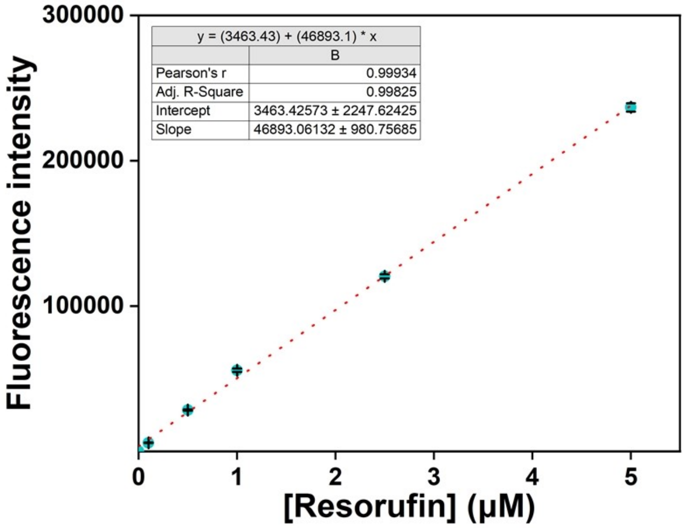

Fig S6. The calibration curve shows the relationship between the fluorescence intensity and the concentration of resorufin.

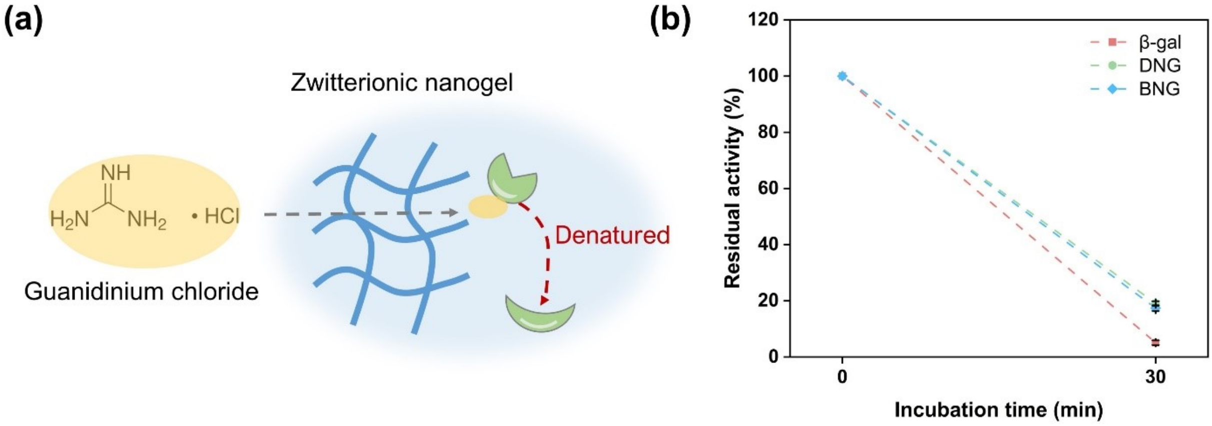

Fig S7. (a) Illustration of the denaturation of β-gal immobilized inside zwitterionic nanogels caused by guanidinium chloride; (b)Residual activity of β-gal, BNG and DNG incubated with 6M guanidinium chloride for 30 min.
